# Muscle Injury Associated Elevated Oxidative Stress and Abnormal Myogenesis in Patients with Idiopathic Scoliosis

**DOI:** 10.7150/ijbs.33340

**Published:** 2019-09-07

**Authors:** Jiong Li, Mingxing Tang, Guanteng Yang, Longjie Wang, Qile Gao, Hongqi Zhang

**Affiliations:** Department of Spine Surgery, Xiangya Hospital, Central South University, No. 87, Xiangya Road, Changsha, Hunan, China, 410008.

**Keywords:** Idiopathic scoliosis, muscle injury, myogenesis, apoptosis, oxidative stress, ROS

## Abstract

Idiopathic scoliosis (IS) is a disease with unknown etiology characterized by spinal rotation asymmetry. Reports describing the histochemical and pathological analyses of IS patients have shown that necrosis, fibrosis and fatty involution occurred on the apex paraspinal muscles. However, research on the changes in the paraspinal muscles of IS patients compared with those in matched controls is rare; thus, the basic mechanism of how paraspinal muscles are injured in IS patients is still unclear. In this study, we investigated the morphological changes of paraspinal muscles in the control group and IS patients, and the possible mechanisms were examined *in vivo* and *in vitro*. Increased myofiber necrosis was found on both sides of the apex paraspinal muscles of IS patients compared with those of the control group, and the number of TUNEL-positive apoptotic cells was also increased. Apoptosis signaling pathways, including pro-apoptosis proteins such as cleaved-caspase 3 and cytochrome c, were markedly upregulated, whereas the anti-apoptotic Bcl-2/Bax was significantly downregulated in IS patients compared with the control group. Moreover, PGC-1α and SOD1 were upregulated in accordance with the increased ROS production in IS patients. The distribution of myofiber types, as well as the mRNA levels of type IIa myofiber marker* MYH2* and the important myogenesis regulator *MYOG* were remarkably changed in IS patients. In addition, C2C12 or human skeletal muscle mesenchymal progenitor cells treated with antimycin A in glucose-free and serum-free culture medium, which can activate oxidative stress and induce apoptosis, showed similar patterns of the changed distribution of myofiber types and downregulation of *MYH2* and *MYOG*. Altogether, our study suggested that the extents of severe muscle injury and accumulated oxidative stress were increased in IS patients compared with the control group, and the abnormal myogenesis was also observed in IS patients. Since elevated oxidative stress can lead to apoptosis and the dysregulation of myogenesis in muscle cells, it may be associated with the pathological changes observed in IS patients and contribute to the development and progression of IS.

## Introduction

Idiopathic scoliosis (IS) is a structural three-dimensional spinal deformity disease with unknown etiology, and its peak incidence is around puberty (adolescents with idiopathic scoliosis, AIS). With the development of disease, the rotation and lateral curve of certain IS patients will progress rapidly, eventually affecting cardiopulmonary and spinal functions, and become life-threatening. IS treatment is costly and incurs a high risk due to its complexity and uncertainty. Therefore, many studies are needed to reveal the pathogeny of IS development and progression.

The maintenance of spinal stability was influenced not only by the spinal column (vertebra, intervertebral disc, facet joint, etc.) but also by the paravertebral muscles and others. The myogenesis process of vertebrate skeletal muscles begins in embryonic muscle progenitor cells followed by proliferation and differentiation, and these precursor cells migrate along an established route to specific sites of new muscle tissue formation and differentiate into skeletal muscles 1. Studies have shown that members of the myogenic regulatory factors family such as myogenin (MYOG) and myoblast determination protein 1 (MyoD1) are important transcription factors that regulate myogenesis 2. Adult skeletal muscle is composed of multinucleated myofibers extending along the length of the muscle. According to the expression of myosin heavy chain (MyHC) isoforms, fiber types are classified as slow‐twitch type I (MyHC I [MYH7]), fast‐twitch oxidative type IIa (MyHC IIa [MYH2]), fast‐twitch oxidative-glycolytic type IIx (mainly expressing MyHC IIx [MYH1]) and fast‐twitch glycolytic type IIb (mainly expressing MyHC IIb [MYH4]) fibers; the first three fibers are the primary categories because type IIb myofibers are apparently absent in normal human muscle 3, 4.

It has been shown that stimulatory muscle forces substantially affect lumbar intradiscal pressure, and a causal relationship between the paravertebral muscles and the spine sagittal alignment was observed 5, 6. Signs of myopathy, muscular atrophy due to necrosis, presence of hyaline fibers, and mitochondrial proliferation were found in the paraspinal muscles of AIS patients 7. Linek, Pawel *et al*. also found that all the muscles on the right side of the body showed higher activity in the AIS group during the active straight leg raise test 8. Yang, Hoe S *et al.* observed a significant difference between the bilateral abdominal muscles in adolescents with IS 9. A recent study has suggested that in AIS patients who have a major curve >30^0^ at skeletal maturity, patients with increased thoracolumbar/lumbar curve progression have decreased skeletal muscle volume and increased fatty degeneration of the lumbar extensor muscles in adulthood 10.

Based upon these observations, it is understood that the status of muscles influences the development of IS and its progression, but we could still not accurately judge whether the changes of paravertebral muscles are the cause of IS. In addition, differences in the morphological changes between the IS patients and the control group were unclear. Studies on the underlying mechanism of paravertebral muscle changes in IS patients are rare. The development of vertebrate skeletal muscles or myogenesis processes in IS patients and whether this process is changed in IS is unknown. Therefore, in this study, we examined the morphological and molecular changes of the paravertebral muscles in IS patients and control subjects. The skeletal muscle myogenesis-related signaling pathway was also evaluated, with the hope of helping to discover the underlying mechanisms of paraspinal muscle histopathology in IS patients.

## Materials and Methods

### Patients and controls

The paravertebral muscle tissues of 33 IS patients and 24 control subjects (non-IS patients with lumbar disc herniation or spine fracture) were collected carefully and harmlessly during the posterior approach surgery in Xiangya Hospital (Table 1 and Table S1). The size of muscle biopsy was about 0.5-1 cm in diameter. Both of the concave and convex side of paraspinal muscles in the IS group were used for the comparative study with the control group. The IS patients and control subjects were identified based on their clinical manifestations, X-ray, CT and MRI results, etc. The exclusion criteria of the patients and controls were as follows: individuals with severe neuromuscular or genetic disease or routinely using hormones or immune inhibitors.

### Histological analysis and pathological evaluation

Fixed muscle samples were embedded in paraffin and cut into 5μm thickness. Sections were stained with hematoxylin-eosin (H&E) as previous 11. Type I (dark staining) and type II (light staining) myofibers were determined by ATPase staining of 8μm thick frozen muscle tissue sections as described with modification 12. After preincubation of 15 minutes (min) in buffer with 200 mM Tris base, 18 mM CaCl_2_, pH 4.3, the sections were incubated for 1 hour (h) in 200 mM Tris base, 18 mM CaCl_2_, 2.75 mM ATP-disodium, pH 9.4. and then rinsed in 3 changes of 1% CaCl_2_ (3 min each); immersed for 3 min in 2% CaCl_2_; and rinsed in 10-12 changes of tap water. After staining for 2 min in 2% (NH4)_2_S the sections were washed with several changes of tap water, dehydrated, cleared and mounted. For analysis, pictures of 4-6 different fields per sample were taken under a Leica DM5000 B microscope equipped with a digital CCD (Leica Camera, Wetzlar, Germany), and semi-quantitative analyses of muscle histology were performed in a blinded fashion.

### TUNEL assay

TUNEL assay (In Situ Cell Death Detection kit, fluorescein; Roche, Mannheim, Germany) was performed to detect apoptotic cell death on the paraffin-embedded retinal sections as manufacturing instructions with some modification 13. TUNEL positive cells were counted at 4-6 different areas per section as described above, and reported as a percentage of TUNEL-positive muscle cells.

### Measurement of Reactive Oxygen Species (ROS)

Muscle tissues were frozen in Tissue-Tek OCT embedding medium (Sakura Finetek, Tokyo, Japan) and cut into 8μm thick sections. 10 μM dihydroethidium (DHE, Beyotime, China) was used to evaluate ROS levels in situ by incubating the slides in a dark chamber at 37 °C for 30 min 14. 2 μM DHE and 10 μM DCFH-DA (ROS Assay Kit, Beyotime, China) were used to incubate the in vitro cells at 37 °C for 20 min for ROS analyses as manufacturing instructions.

### NO assay

Muscle tissues and cells were homogenized, the supernatant after centrifugation was saved for NO analysis. Two microliters of supernatant was removed for protein quantification with bicinchoninic acid reagent following the manufacturer's instructions (KWBIO, Beijing, China). NO levels were detected by a Griess reaction based on measuring the concentration of nitrite and nitrate using a NO assay kit (Beyotime, Shanghai, China) according to the manufacturer's instructions. NO data were expressed as nmoles per milligram of protein 15.

### Extraction of primary skeletal muscle mesenchymal progenitor cells (hSM-MPC) and Cell experiment

The hSM-MPC were isolated enzymatically according to the protocols described 16. Briefly, mechanically disrupted paravertebral muscle from 24y old male lumbar disc herniation patients were digested by 0.1% collagenase I (Sigma-Aldrich, Germany) for 60 min at 37°C. And then, the cell suspension was centrifuged for 5 min at 1000 × g, the pellet was resuspended by high glucose DMEM (Hyclone, Logan, USA) supplemented with 10% horse serum. Settle the resuspended fibers for 5 min and transfer the supernatant containing stem cells to a fresh tube. The collected supernatant was filtered through a 40 μm nylon cell strainer and centrifuged for 10 min at 1000 × g, the pellet of cells was cultured in growth medium (high glucose DMEM supplemented with 10% FBS and 1% penicillin-streptomycin) at 37 °C, as well as C2C12 myoblasts. And Paired box 7 and Desmin immunofluorescent staining was used for hSM-MPC identification before and after differentiation respectively (Sup Fig. 1). Fixed cells were blocked by 5% bovine serum albumin with 0.3% Trion X-100 at room temperature (RT) for 30 min, and then were incubated with primary antibodies (Santa Cruz Biotechnology, Dallas, USA) respectively for overnight at 4 °C. After extensive washing, sections were incubated with fluorescein-labeled anti-rabbit immunoglobulin G (Servicebio, Wuhan, China) for 2h at RT. Cells were co-stained with DAPI (Servicebio).

Before antimycin A stimulation, human skeletal muscle mesenchymal progenitor cells or C2C12 myoblasts were shifted to differentiation medium (high glucose DMEM containing 2% horse serum and 1% penicilin-streptomycin) 17. No glucose and no serum DMEM medium with 100nM antimycin A (AA, Abcam, Cambridge, UK) or differentiation medium with DMSO (preparation of AA stock solutions) were used for 1h stimulation and then changed back to normal differentiation medium. Cells were harvested 2h later for experiments respectively.

### Apoptosis analysis by annexin V staining

Apoptosis was measured by FITC Annexin V Apoptosis Detection Kit I (BD Biosciences, San Jose, CA) according to the manufacturer's instructions. Briefly, 5 µl of FITC Annexin V and 5 µl PI were added for each 100 µL cell suspension and incubated for 20 min in a dark place at RT, and then analyzed by flow cytometry within 1h 18.

### Western Blotting

Muscle tissues and cells were sonicated in RIPA buffer (Beyotime Biotechnology, Jiangsu, China) to obtain whole cell lysates. Western blot analysis was performed as previously reported 19. Antibodies for cleaved-caspase 3 and endothelial NO synthases (eNOS) was obtained from Cell Signaling Technologies (Danvers, MA), antibodies for B cell leukemia/lymphoma 2 (Bcl2), BCL2-associated X protein (Bax) and glyceraldehyde-3-phosphate dehydrogenase (GAPDH) were from Proteintech Group, Inc (Chicago, USA) while the remaining antibodies were purchased from Servicebio (Wuhan, China). The expression levels of target proteins were quantified with the Quantity One 1-D Analysis Software (Bio-Rad) and normalized to the GAPDH in the same sample.

### Real time PCR

Total RNA for muscle tissues and cells was extracted using TRIzon Reagent (CWBiotech, Beijing, China). Equal mass of RNA of each sample was reverse transcribed into cDNA using HiFiScript cDNA Synthesis Kit (CWBiotech, Beijing, China). The real time PCR was performed with specific primers for the targeted genes by NovoStart® Probe qPCR Super Mix (Novoprotein, Shanghai, China) and the transcriptional levels were quantified using GAPDH in the same sample as internal control. The sequences of primers used are described in Table 2 and 3.

### Statistical analysis

All results were expressed as mean ± SD (Standard Deviation). The combined results of two sides of paraspinal muscle in the IS group or control group were used for statistical analysis. Statistical significance was mainly determined by the Student's *t*-test except the sex difference was calculated by *x*^2^ test. Differences were considered statistically significant when *p* <0.05.

## Results

### General physiological features of IS patients and control groups

As shown in Table 1, a population of 24 controls and 33 IS patients were studied in our research, and the mean ages of the two groups were 17.23 years and 15.80 years, respectively, with no significant difference. There were 15 females and 9 males in the control group, while there were 19 females and 14 males in the IS patient group. No sex difference between the two groups was observed according to a *x*^2^ test (Table 1). In addition, the IS patients had an average Cobb angle of 47.71 degrees (Table 1).

### Severe paravertebral muscle injury in IS patients

**Fig 1:**
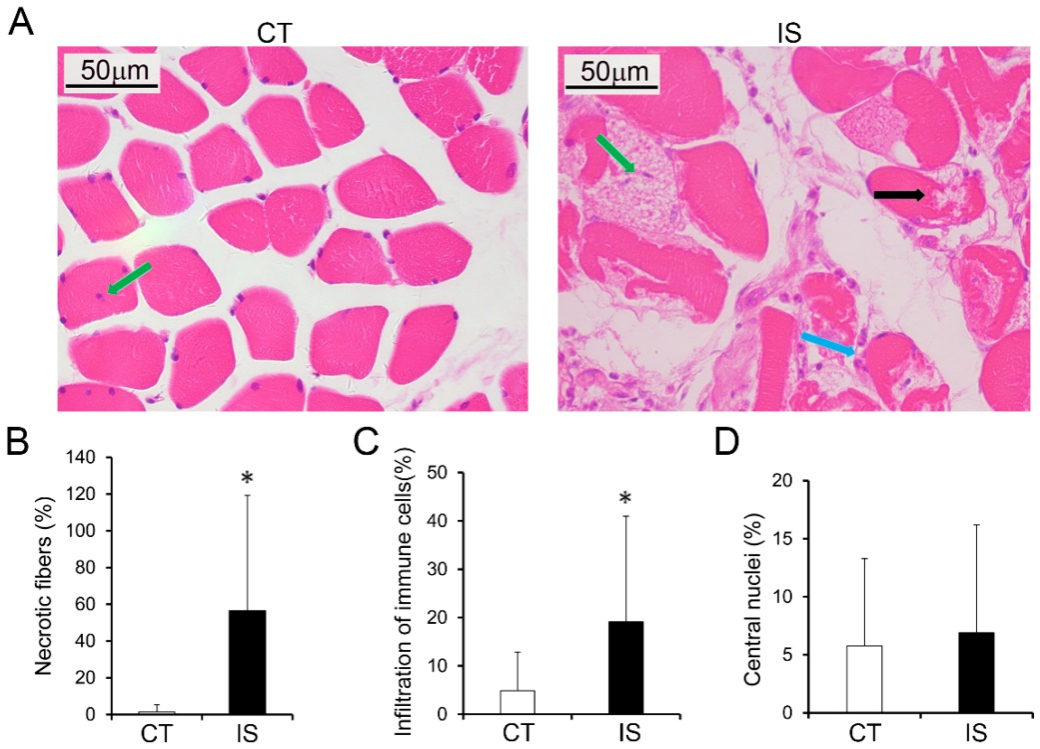
Histopathological analysis of paraspinal muscles in IS patients and control group. H&E stained paraspinal muscle sections of IS patients and control group (A); black arrow, necrosis; blue arrow, infiltration of immune cells; green arrow, central nuclei. B-D, Quantity evaluation of muscle injury, percentage ratio of infiltration of immune cells to total cells, and percentage of central nuclei and. n=15-20, *p<0.05.

The histopathological features of the paravertebral muscles in the apex vertebral region of IS patients and the non-diseased region of the control group were analyzed. As shown in Fig. 1A, H&E staining results suggested that relatively normal myofibers and neither necrosis nor infiltration of immune cells were observed in the paravertebral muscles of the control group. However, in the IS patients, marked necrosis of myofibers was found in both the concave and convex sides of the apex vertebral muscles, although there was no significant difference between the two sides (Fig. 1B and Sup Fig. 2). In addition, the infiltration of immune cells was much more increased in the paravertebral muscles of IS patients than controls (Fig. 1C). Even fibrosis, could be observed in some IS patients (data not shown). Surprisingly, very few central nuclei were observed in both the IS patients and the control group (Fig. 1D).

**Fig 2:**
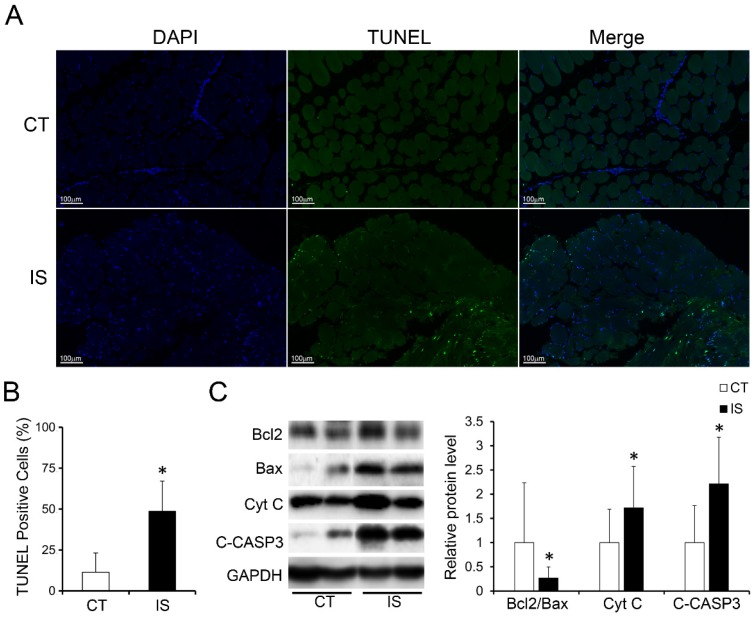
Increased apoptosis in paraspinal muscles of IS patients. A, TUNEL stained paraspinal muscle sections of IS patients and control group, 200×, n=15-20. B, Quantity evaluation of percentage of TUNEL positive muscle cells, n=15-20. C, WB analysis of apoptosis related proteins and quantification results. C-CASP3, cleaved-caspase 3, n=24-33. *p<0.05.

In addition, much more apoptosis was discovered in the paravertebral muscles of the IS patients than those in the control group according to the TUNEL staining results, which showed that the proportion of TUNEL-positive cells was significantly increased in the IS patients (Fig. 2A-B). Similar to the H&E staining results, the proportion of TUNEL-positive cells between the concave and convex sides of the apex vertebral muscles in the IS patients was not significantly different (Sup Fig. 3).

Since the remarkable muscle injury changes between the IS patients and control people were observed, whereas little difference between the concave and convex sides of IS patients was found, our subsequent experiments focused on the differences between the IS patients and the control group rather than on the two sides of IS patients.

### Apoptosis-related pathways were dysregulated in the paravertebral muscles of IS patients

We further investigated the expression levels of apoptosis-related proteins in the paravertebral muscle of the IS patients and control group. In accordance with the TUNEL staining results, pro-apoptosis proteins such as cytochrome c (Cyt C) and cleaved-caspase 3 were markedly increased in IS patients compared with the control group (Fig. 2C). Moreover, the expression ratio of Bcl-2/Bax, an anti-apoptosis marker, was significantly decreased in IS patients compared with the control group (Fig. 2C). These data showed that the increased apoptosis in the paravertebral muscles of IS patients may result from the upregulation of pro-apoptosis proteins and the downregulation of anti-apoptosis proteins.

### Elevated oxidative stress in the paravertebral muscles of IS patients

**Fig 3:**
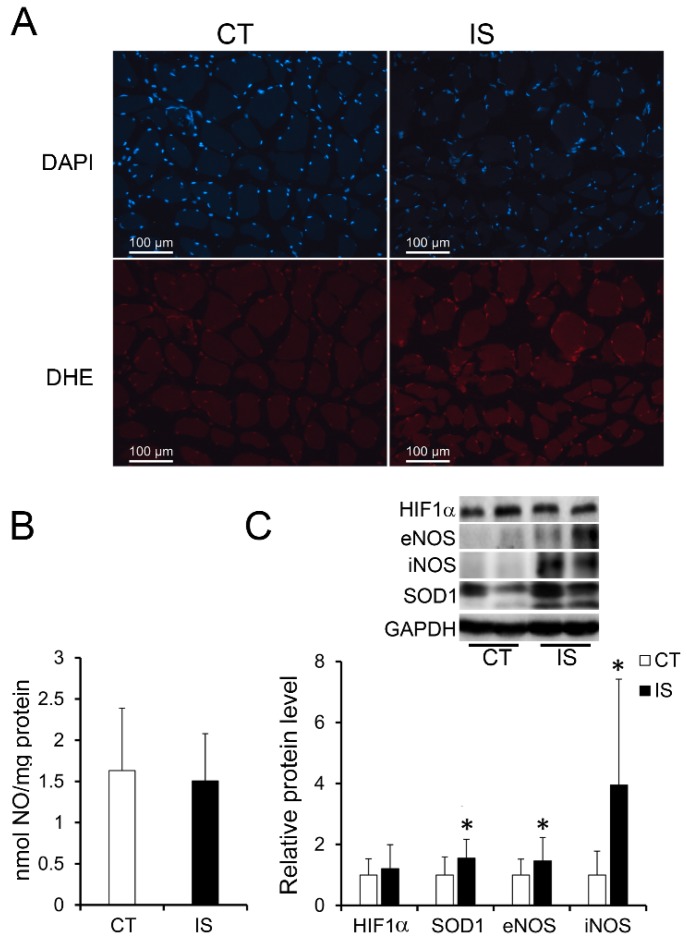
Elevated oxidative stress in paraspinal muscles of IS patients. A, DHE stained superoxide anion production of IS patients and control group, n=9-13. B, The concentration of NO evaluated by a Griess reaction in IS patients and control group respectively, n=24-33. C, The expression levels of oxidative stress related proteins, n=24-33. *p<0.05.

It has been reported that the oxidative stress-induced production of ROS and reactive nitrogen species (RNS) in skeletal muscle is associated with apoptosis and necrosis 20, 21. Therefore, we further evaluated the production of ROS and RNS. As shown in Fig. 3A, after staining with DHE, which can be mainly oxidized by superoxide anion to a product with red fluorescence, the paravertebral muscle of IS patients showed stronger red fluorescence than the control group, suggesting an increase in ROS levels in IS patients. However, the concentrations of NO (one major RNS) in the IS patients and the control group showed no significant difference (Fig. 3B). Surprisingly, NO synthases such as eNOS and inducible NO synthases (iNOS) were significantly increased in the paravertebral muscles of IS patients compared with those of the control group; SOD1, which is a main antioxidant enzyme that catalyzes the superoxide anion into H_2_O_2_, was also significantly increased in the paravertebral muscles of IS patients. (Fig. 3C). However, there were no significant changes in the protein levels of hypoxia-inducible factor 1α (HIF-1α) in the IS patients and the control group (Fig. 3C). The results suggest that oxidative stress may be elevated in the paravertebral muscles of IS patients compared with those of the control group in a hypoxia-independent manner.

### Changed myofiber types and expression of MyHC genes in the paravertebral muscles of IS patients

**Fig 4:**
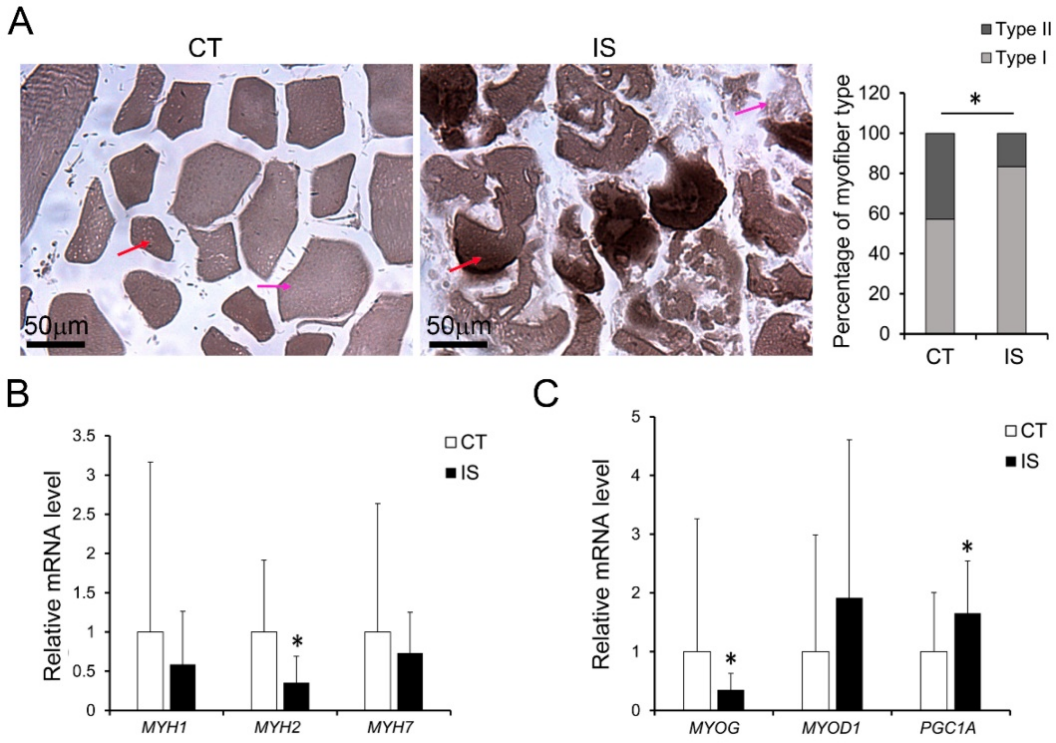
Changes of myogenesis in paraspinal muscles of IS patients and control group. A, ATPase staining results of IS patients and control group, n=9-13. The mRNA levels of MyHC isoforms (B) and myogenesis related genes (C) in IS patients and control group, n=24-33. *p<0.05.

As oxidative stress plays important roles not only in skeletal muscle physiology but also in myofiber adaptation 22, we also examined the myofiber types and gene expression of MyHC isoforms in both the IS patients and control group. As shown in Fig. 4A, the percentage distribution of light stained type II myofibers and dark stained type I myofibers were significantly changed in IS patients compared with controls, but little difference was found between concave and convex sides in IS patients (Sup Fig. 4). Meanwhile, the mRNA level of *MYH2*, which is a marker of type IIa myofibers, was dramatically decreased in IS patients (Fig. 4B). Similarly, the mRNA level of *MYOG* was markedly decreased in IS patients (Fig. 4C). However, there was no significant difference in the mRNA levels of *MYH1*, *MYH7* and *MYOD1* between the IS patients and the control group (Fig. 4B-C). In contrast, the mRNA level of peroxisome proliferator-activated receptor γ coactivator-1 A (*PGC1A*), which can drive the transition of fast-twitch fiber into slow-twitch myofibers and play an important role in oxidative stress, was increased in IS patients (Fig. 4C).

Together, these results showed that abnormal oxidative stress may accumulate more in the paravertebral muscles of IS patients than those of the control group, and unrelieved oxidative stress may further cause necrosis, apoptosis, and the dysregulation of myogenesis in the paravertebral muscles of IS patients.

### Oxidative stress induced myofiber injury in C2C12 cells or hSM-MPC

**Fig 5:**
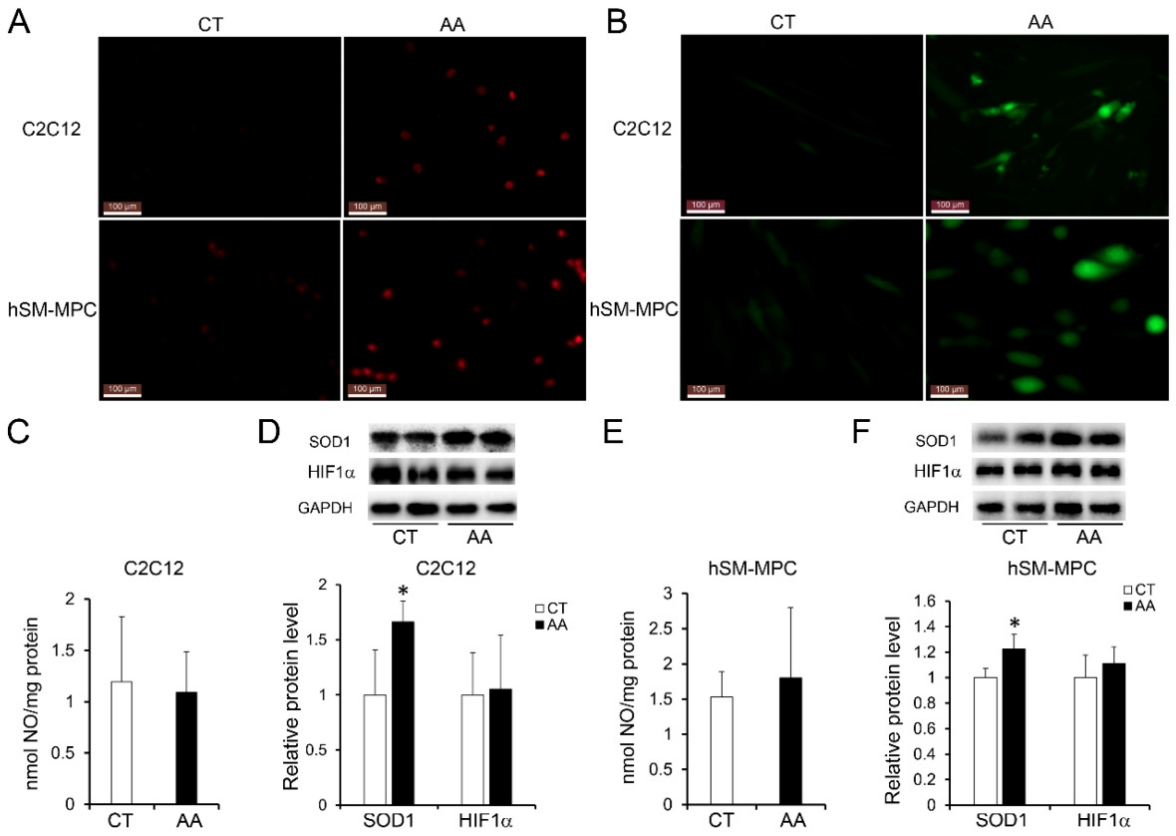
Elevated oxidative stress in AA treated C2C12 cells or hSM-MPC. A, DHE stained superoxide anion production of AA treated and control cells. B, ROS production of AA treated and control cells. The concentration of NO evaluated by a Griess reaction in AA treated and control cells respectively (C, E). The expression levels of oxidative stress related proteins (D, F). hSM-MPC, human skeletal muscle mesenchymal progenitor cells. n=6, *p<0.05.

To test the impact of increased oxidative stress on myofiber pathology, we treated differentiated C2C12 cells (the mouse myoblasts commonly used as an in vitro system to study myogenesis process) or hSM-MPC with AA, which can inhibit the mitochondrial electron transport chain from cytochrome B to Cyt C and lead to ROS production 23. According to the ROS assay results, AA-treated differentiated C2C12 cells and hSM-MPC both showed increased ROS production compared with their control cells (Fig. 5A-B). Similar to the observation of the paravertebral muscles in the IS patients and the control group, NO production did not significantly change in AA-treated two group cells (Fig. 5C, E). The protein levels of SOD1 were significantly increased in the AA group compared with the control group, while the expression of HIF-1α was not different (Fig. 5D, F).

**Fig 6:**
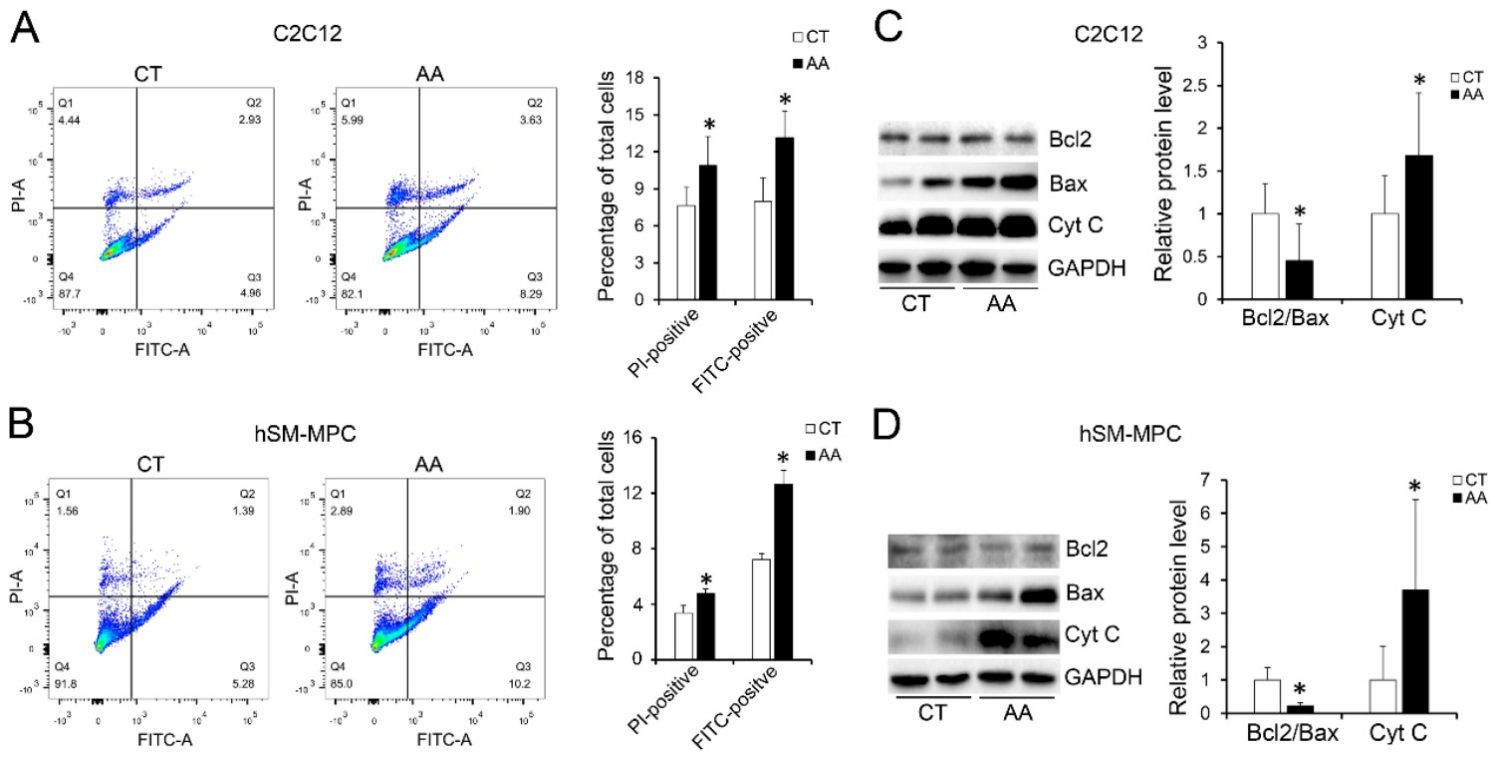
Increased apoptosis in AA treated C2C12 cells or hSM-MPC. A-B, flow cytometry analysis of FITC Annexin V and PI stained cells in AA treated and control cells and the statistical analysis. C-D, WB analysis of apoptosis related proteins and quantification results. hSM-MPC, human skeletal muscle mesenchymal progenitor cells. n=6, *p<0.05.

**Fig 7:**
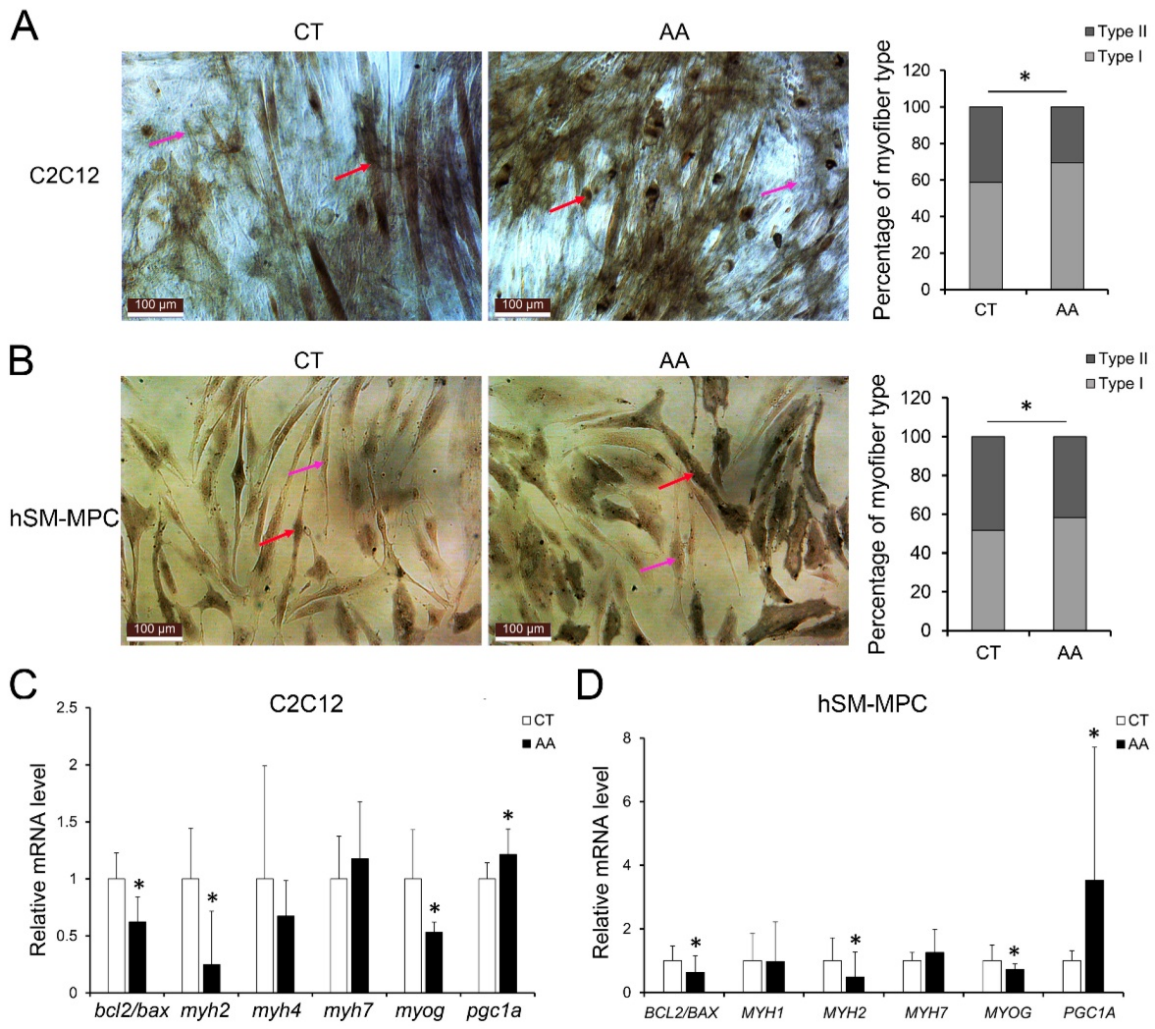
Abnormal myogenesis in AA treated C2C12 cells or hSM-MPC. A, ATPase staining results of AA treated and control cells. B-C, The mRNA levels of apoptosis, MyHC isoforms and myogenesis related genes. hSM-MPC, human skeletal muscle mesenchymal progenitor cells. n=6, *p<0.05.

Furthermore, apoptosis assays showed increased percentage of PI-positive cells and FITC-positive cells, which indicated more apoptosis or necrosis in the two AA groups compared with their control groups (Fig. 6A-B). The decreased expression of Bcl-2/Bax at both the protein and mRNA levels, together with the increased expression of the pro-apoptosis protein Cyt C were also observed in the two AA groups compared with their control groups respectively (Fig. 6C-D). Meanwhile, ATPase staining results of C2C12 cells or hSM-MPC showed changed percentage distribution of myofibers types after AA treatment (Fig. 7A-B). Moreover, the mRNA levels of *MYH2* and *MYOG* were significantly decreased, while *PGC1A* was significantly increased in AA-treated C2C12 cells or hSM-MPC (Fig. 7C-D). However, there was no significant difference in the mRNA levels of *myh4* or *MYH1* and *myh7/MYH7* between the AA groups and their controls (Fig. 7C-D).

The changes we observed in the C2C12 cells or hSM-MPC with an AA-induced elevation in oxidative stress may suggest that myofiber injury in the paravertebral muscles of IS patients was largely related to unrelieved oxidative stress.

## Discussion

The etiology and pathogenesis of IS, a spinal deformity disease with a prevalence of approximately 0.5-5% worldwide, is still unclear 24-26. To date, various theories, such as genetics, structural and environmental factors, have been proposed by scientists 27. In addition to vertebral bone distortions, paraspinal muscle disorder was considered one of the most likely primary structural causes for IS 7. The paraspinal muscles mainly include the psoas (PS), erector spinae (ES) and multifidus (MF), which play important roles in spinal stability 6, 28. Therefore, massive abnormalities in these muscles of IS patients were reported. Increased type I fibers on the convex side and decreased type II fibers on the concave side of the curvature were observed in IS patients 29. Furthermore, dramatic morphological changes such as hypertrophy, atrophy, centralized nuclei, and disrupted myofibrillar elements were detected in some muscle cells on the concave aspect of the curve 30. And Wajchenberg *et al.* showed that the paraspinal muscles on the concave side of the scoliosis apex had significantly more fibrosis and fatty involution than those on the convex side 7.

However, all of these studies are detailed analyses of the concave and convex sides in the paraspinal muscle of IS patients, and the fundamental problem of how paraspinal muscle is injured in IS patients remains controversial because of the lack of a matched control for the comparative analysis of paraspinal muscle changes. In view of the lack of a good animal model exhibiting the anatomical features of the rotatory spinal deformity observed in IS, human muscle biopsies were primarily used in our study. Similar to previous studies, we also found increased levels of necrosis and infiltration of immune cells on both sides of the apex vertebra of IS patients compared with those of the matched control groups, although there were no significant differences in the extent of necrosis or apoptosis of the two sides of the paraspinal muscle (Fig. 1-2 and Sup Fig.2-3). In parallel with the percentage distribution of myofiber types, which showed increased percentage of type I myofibers while decreased percentage of type II myofibers in IS patients, the loss of type IIa fibers number (*MYH2*) was also observed in IS patients compared with the control group in our study (Fig. 4). Nevertheless, type I fibers number (*MYH7*) was not increased in IS patients compared with the control group (Fig. 4). These results may be related to the overall myofibers loss in IS patients after injury.

It is well demonstrated that skeletal muscle has a high capacity to adapt to various physiological environments, and this adaptation is possible because of the properties of different types of muscle fibers 22. Therefore, the kinds of muscle fiber adaptation observed in our study may indicate that the physiological microenvironments around the paraspinal muscles have been changed.

In addition to the adaptation, the skeletal muscle is also capable of functional recovery after injury 31. Ordinarily, the restoration of damaged skeletal muscle function requires the balance between regenerative capacity and apoptosis rate 32, 33. Apoptosis is a process that is essential to development and to tissue homeostasis, but it is also pathogenic 34. We found severely increased apoptosis in the paraspinal muscles of IS patients, on both the concave and convex sides, compared with those of the control group (Fig. 2). Together with the severe muscle injury observed in IS patients, it is likely that the balance between the regeneration and apoptosis in the paraspinal muscles of IS patients has been disrupted and pathogenic apoptosis occurs. Moreover, the centralization of nuclei, which occurred after muscle injury and regeneration, was only observed in a few muscle cells of both the IS patients and the control group, and there was no difference between the groups (Fig. 1). These findings may further suggest that muscle regeneration may fail after severe muscle injury in IS patients. In general, skeletal muscle regeneration requires the activation and differentiation of satellite cells (SCs), which are adult muscle stem cells 35, 36. Therefore, the failure of muscle regeneration may be related to insufficient SC activation. To date, research on the physiological changes of SCs in the paraspinal muscle of IS patients has not been conducted. Whether the number of SCs in IS patients has changed or the activation and differentiation of SCs are blocked is unclear, and related investigations are needed.

Mitochondrial dysfunction has been implicated in many skeletal muscle pathologies, including aging-induced atrophy and loss of type II myofibers 37. The plasticity of mitochondria is a key factor in the cellular metabolic adaptation to exercise or the inactivity of skeletal muscle 38. The transcriptional coactivator PGC-1α, which can activate mitochondrial biogenesis and oxidative metabolism, is reported to drive the formation of slow-twitch and type IIx muscle fibers 39-41. In addition, PGC-1α buffers the oxidative stress occurring during muscle cell differentiation by promoting the expression of antioxidant enzymes, and the downregulation of PGC-1α led to the impairment of antioxidant gene expression accompanied by an oxidative burst 42. Generally, ROS/RNS play a dual role since they can be either harmful or beneficial to living systems. Oxidative stress ensues when a greater imbalance between ROS/RNS generation and antioxidant system activity occurs in favor of the ROS/RNS 21, 43, 44. In our study, PGC-1α was significantly upregulated in IS patients, which may result from increased ROS production and could be associated with the changed percentage distribution of myofiber types and the dramatic decrease of *MYH2* rather than *MYH1* and *MYH7,* as we found similar results in AA-treated C2C12 cells and hSM-MPC (Fig. 4-7). Thus, we hypothesize that the PGC-1α-induced expression of antioxidant enzymes may be insufficient to eliminate the elevated oxidative stress, which could further lead to abnormal myogenesis and aggravate the muscle injury we observed in IS patients.

Interestingly, there were no significant changes in the NO production of the IS patients and the control group, and AA-treated C2C12 cells and hSM-MPC also did not show a difference in NO production from control cells. However, the protein levels of iNOS and eNOS were both upregulated in IS patients compared with the control group (Fig. 3&5). This could be because we tested the production of NO indirectly through its stable final substrate. Nevertheless, ROS may have an important role in the pathological changes of the paravertebral muscles in IS instead of RNS, given that eNOS may shift from producing NO to producing superoxide anion by S-glutathionylation modifications 45. However, further investigation may also be integral to determining the changes in RNS production and the exact roles of enzymes related to this process.

Different from the previous studies which focused on the difference between the concave and convex sides of the paraspinal muscle in IS patients, our study aimed at comparative analysis of paraspinal muscle changes between the matched control subjects and IS patients. Our data suggested the increased unresolved oxidative stress in IS patients compared with control subjects may affect the process of myogenesis and induce the necrosis and apoptosis of muscle cells. However, the first cause of the oxidative stress elevating in IS patients and its exact role in the development or progression of IS cannot be verified directly in our study, so that well-designed animal studies are still needed. Furthermore, once the spinal curve was formed, the stress that the cells from the concave and the convex side endured would become different, the muscle injury of the two sides may become different as well. The difference could further lead to the increase of deformity on one hand, on the other hand, this difference would widen with the degree of curve. Therefore, the concavity would become more affected in more severe patients. These may be the reasons that differences of myofiber types proportion or fibrosis or fatty involution between the concave and convex sides were observed in the studies of Wajchenberg *et al.* and others while no significant changes were found in our study since the mean deformity of the patients in their study was higher. Although no significant changes in injury of muscle tissues from different sites was observed in our study (data not shown), and the ES between thoracic and lumbar regions did not differ significantly in their percentage distribution of myofiber types, the ratio between type I and II fibers varied only slightly in the different locations of the muscle group 46, 47, it was better to withdraw the muscle from the same site as the IS patients in control subjects for etiologic studies since different muscle fiber types are characterized with different levels of mitochondrial activity and oxidative stress.

**Fig 8:**
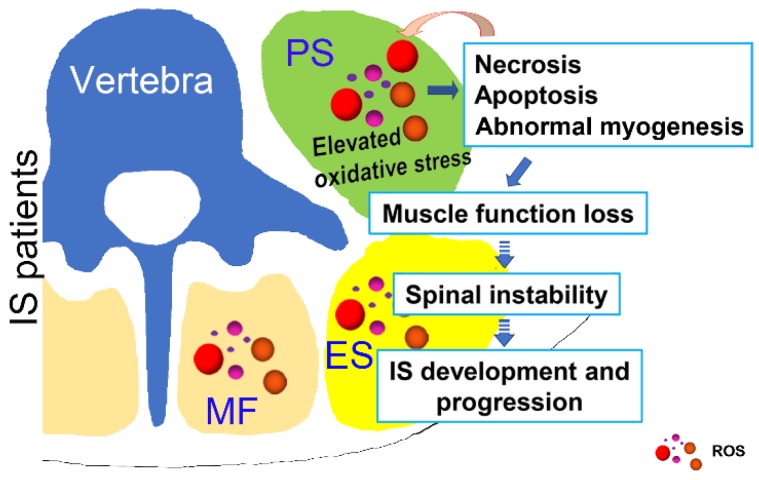
Possible muscle injury related under mechanism in the development of IS. Elevated oxidative stress in paravertebral muscles may lead to necrosis, apoptosis and abnormal myogenesis and further impact the development or progression of IS. PS: psoas; ES: erector spinae; MF: multifidus; ROS: reactive oxygen species.

In conclusion, as shown in Fig. 8, our study showed that unresolved oxidative stress can impact the process of myogenesis and induce the necrosis and apoptosis of muscle cells, which can subsequently increase the production of ROS. Such changes may further lead to the loss of muscle function and impair spinal stability, ultimately contributing to the development and progression of IS. Our results may suggest an important role of oxidative stress in the development and progression of IS, which improves our understanding of the etiopathogenesis of IS and offers a new target for a more effective IS therapy.

## Supplementary Material

Supplementary figures and table.

## Figures and Tables

**Table 1:** Demographic of study populations

Characteristics	CT *N*=24	IS *N*=33	*P* value
Age (years)	17.23 ± 4.27	15.80 ± 3.07	0.074
Sex	F 15 / M 9	F 19 / M 14	0.461
Major curve Cobb Angle (^o^)	‫—	47.71 ± 15.41	—

CT, control group; IS, idiopathic scoliosis patients; F, female; M, male. Data was present as Mean ± Standard Deviation.

**Table 2:** Primers sequences for human genes

GENE	Primer sequence (5'-3')	Length (bp)
*MYH1*	F: AAGAAGCGGAGGAACAATC	187
	R: TGGTCACCTTTCAGCAGTTAG	
*GAPDH*	F: TGACCCCTTCATTGACCTCA	166
	R: ATCGCCCCACTTGATTTTGG	
*MYH2*	F: TCTCAGGCTTCAAGATTTGG	187
	R: CTTCACCCGCAGTTTGTTCA	
*PGC1A*	F: GCTGTACTTTTGTGGACGCA	199
	R: AGGTATTCGCCATCCCTCTG	
*MYH7*	F: GACGGAGGAGGACAGGAAAA	246
	R: TCCTCATTCAAGCCCTTCGT	
*MYOG*	F: TGGACAGCATCACAGTGGAA	209
	R: GAGGAAGGGGATAGTCTGGC	
*MYOD1*	F: TATGGAGCTACTGTCGCCAC	189
	R: GAGTGCTCTTCGGGTTTCAG	

Abbreviation: F, Forward; R, Reverse.

**Table 3:** Primers sequences for mouse genes

GENE	Primer sequence (5'-3')	Length (bp)
*myh2*	F: AGGCAGAGGAGGACAAAGTC	158
	R: CTGGGCTAACTTCAAGTCGC	
*gapdh*	F: CCCACTCTTCCACCTTCGAT	181
	R: CTTGCTCAGTGTCCTTGCTG	
*myh4*	F: AGGGAATGCTGAAGGACACA	161
	R: TCTCCTGCTCCTCTCTGTCT	
*bcl2*	F: CACACACACACATTCAGGCA	154
	R: GGCAATTCCTGGTTCGGTTT	
*bax*	F: GGATGATTGCTGACGTGGAC	175
	R: ATGGTTCTGATCAGCTCGGG	
*pgc1a*	F: TCAGAACCATGCAGCAAACC	177
	R: TTGGTGTGAGGAGGGTCATC	
*myh7*	F: GTGCCAATGACGACCTGAAA	234
	R: CTGTCTGGAGCTGGGATAGG	
*myog*	F: CGCCATCCAGTACATTGAGC	170
	R: GACCGAACTCCAGTGCATTG	

Abbreviation: F, Forward; R, Reverse.

## Abbreviations

IS: idiopathic scoliosis
AIS: adolescents with idiopathic scoliosis
MYOG: myogenin
MYOD1: myoblast determination protein 1
MyHC: myosin heavy chain
H&E: hematoxylin-eosin
min: minutes
h: hours
ROS: reactive oxygen species
DHE: dihydroethidium
hSM-MPC: human skeletal muscle mesenchymal progenitor cells
RT: room temperature
AA: antimycin A
eNOS: endothelial NO synthases
Bcl2: B cell leukemia/lymphoma 2
Bax: BCL2-associated X protein
GAPDH: glyceraldehyde-3-phosphate dehydrogenase
SD: Standard Deviation
CytC: cytochrome c
RNS: reactive nitrogen species
iNOS: inducible NO synthases
HIF-1α: hypoxia-inducible factor 1α
PGC1A: peroxisome proliferator activated receptor γ coactivator-1 A
PS: psoas
ES: erector spinae
MF: multifidus
SCs: satellite cells
