# Supplementary material for: Muscle Injury Associated Elevated Oxidative Stress and Abnormal Myogenesis in Patients with Idiopathic Scoliosis
Source: Int J Biol Sci. 2019 Sep 7;15(12):2584–95. doi: 10.7150/ijbs.33340 (PMC6854377; doi:10.7150/ijbs.33340)
Supplement: Supplementary file 1 — Supplementary figures and table. [file ijbsv15p2584s1.pdf]

Supplemental materials

Table S1. Clinical data of study subjects

Group	Age	Gender	Major curve Cobb Angle (°)	Lenke Type	Cause of the surgical intervention	Location	Use of muscle tissues
C1	18	F	-	-	Lumbar disc herniation	L4	WPL/RNA/Paraffin Section
C2	18	F	-	-	Lumbar disc herniation	L4	WPL/RNA/Paraffin Section
C3	16	F	-	-	Lumbar disc herniation	L4	WPL/RNA/Paraffin Section
C4	20	F	-	-	Traumatic lumbar disc herniation	L5	WPL/RNA/Paraffin Section
C5	28	F	-	-	Lumbar disc herniation	L4	WPL/RNA/Paraffin Section
C6	17	F	-	-	Lumbar disc herniation	L4	WPL/RNA/Paraffin Section
C7	24	F	-	-	Lumbar disc herniation	L5	WPL/RNA/Frozen Section
C8	15	F	-	-	Lumbar disc herniation	L5	WPL/RNA/Paraffin Section
C9	14	F	-	-	Lumbar disc herniation	L4	WPL/RNA/Paraffin Section
C10	18	F	-	-	Lumbar disc herniation	L5	WPL/RNA/Paraffin Section
C11	10	F	-	-	Lumbar disc herniation	L4	WPL/RNA/Paraffin Section
C12	11	F	-	-	Lumbar disc herniation	L4	WPL/RNA/Frozen Section
C13	10	F	-	-	Lumbar disc herniation	L5	WPL/RNA/Frozen Section
C14	20	F	-	-	Lumbar disc herniation	L5	WPL/RNA/Frozen Section
C15	18	F	-	-	Lumbar disc herniation	L5	WPL/RNA/Frozen Section
C16	19	M	-	-	Lumbar disc herniation	L4	WPL/RNA/Paraffin Section
C17	13	M	-	-	Traumatic lumbar disc herniation	L2	WPL/RNA/Paraffin Section
C18	19	M	-	-	Lumbar disc herniation	L3	WPL/RNA/Paraffin Section
C19	17	M	-	-	Lumbar disc herniation	L4	WPL/RNA/Paraffin Section
C20	18	M	-	-	Lumbar disc herniation	L4	WPL/RNA/Paraffin Section
C21	22	M	-	-	Lumbar disc herniation	L1	WPL/RNA/Frozen Section
C22	16	M	-	-	Lumbar disc herniation	L4	WPL/RNA/Frozen Section

Group	Age	Gender	Major curve Cobb Angle (º)	Lenke Type	Cause of the surgical intervention	Location	Use of muscle tissues
C23	20	M	-	-	Lumbar disc herniation	L4	WPL/RNA/Frozen Section
C24	13	M	-	-	Lumbar disc herniation	L4	WPL/RNA/Frozen Section
IS1	17	F	55	4	Scoliosis	T10	WPL/RNA/Frozen Section
IS2	16	F	27.6	5	Scoliosis	L1	WPL/RNA/Frozen Section
IS3	19	F	40	5	Scoliosis	L1	WPL/RNA/Frozen Section
IS4	15	F	48.6	4	Scoliosis	L2	WPL/RNA/Frozen Section
IS5	21	F	37	5	Scoliosis	L2	WPL/RNA/Frozen Section
IS6	12	F	49.1	1	Scoliosis	T8	WPL/RNA/Frozen Section
IS7	18	F	43.1	1	Scoliosis	T10	WPL/RNA/Paraffin Section
IS8	14	F	89.6	3	Scoliosis	L3	WPL/RNA/Paraffin Section
IS9	12	F	34	5	Scoliosis	L2	WPL/RNA/Frozen Section
IS10	12	F	36.1	5	Scoliosis	L2	WPL/RNA/Paraffin Section
IS11	29	F	42.8	2	Scoliosis	T9	WPL/RNA/Paraffin Section
IS12	16	F	68.1	4	Scoliosis	L1	WPL/RNA/Paraffin Section
IS13	14	F	49.7	1	Scoliosis	T8	WPL/RNA/Paraffin Section
IS14	14	F	52	1	Scoliosis	T9	WPL/RNA/Paraffin Section
IS15	15	F	38	5	Scoliosis	L2	WPL/RNA/Paraffin Section
IS16	16	F	36.7	1	Scoliosis	T8	WPL/RNA/Paraffin Section
IS17	13	F	40.5	1	Scoliosis	T8	WPL/RNA/Paraffin Section
IS18	14	F	62.2	1	Scoliosis	T10	WPL/RNA/Paraffin Section
IS19	17	F	38.9	5	Scoliosis	L1	WPL/RNA/Paraffin Section
IS20	15	M	38.6	1	Scoliosis	T9	WPL/RNA/Frozen Section
IS21	16	M	74	2	Scoliosis	T9	WPL/RNA/Frozen Section
IS22	16	M	28	1	Scoliosis	T9	WPL/RNA/Frozen Section
IS23	14	M	79	2	Scoliosis	T9	WPL/RNA/Frozen Section

Group	Age	Gender	Major curve Cobb Angle (º)	Lenke Type	Cause of the surgical intervention	Location	Use of muscle tissues
IS24	15	M	38.3	5	Scoliosis	L1	WPL/RNA/Frozen Section
IS25	18	M	32.1	5	Scoliosis	L1	WPL/RNA/Frozen Section
IS26	16	M	37.2	5	Scoliosis	L1	WPL/RNA/Paraffin Section
IS27	16	M	75	4	Scoliosis	L2	WPL/RNA/Paraffin Section
IS28	14	M	43.6	1	Scoliosis	T7	WPL/RNA/Paraffin Section
IS29	16	M	55.8	2	Scoliosis	T5	WPL/RNA/Paraffin Section
IS30	16	M	34.6	5	Scoliosis	T12	WPL/RNA/Paraffin Section
IS31	15	M	36.8	1	Scoliosis	T9	WPL/RNA/Paraffin Section
IS32	14	M	54.3	1	Scoliosis	T9	WPL/RNA/Paraffin Section
IS33	15	M	58	4	Scoliosis	L2	WPL/RNA/Paraffin Section

C, control subjects; IS, idiopathic scoliosis; L, Lumbar; T, thoracic, WPL, whole protein lysates.

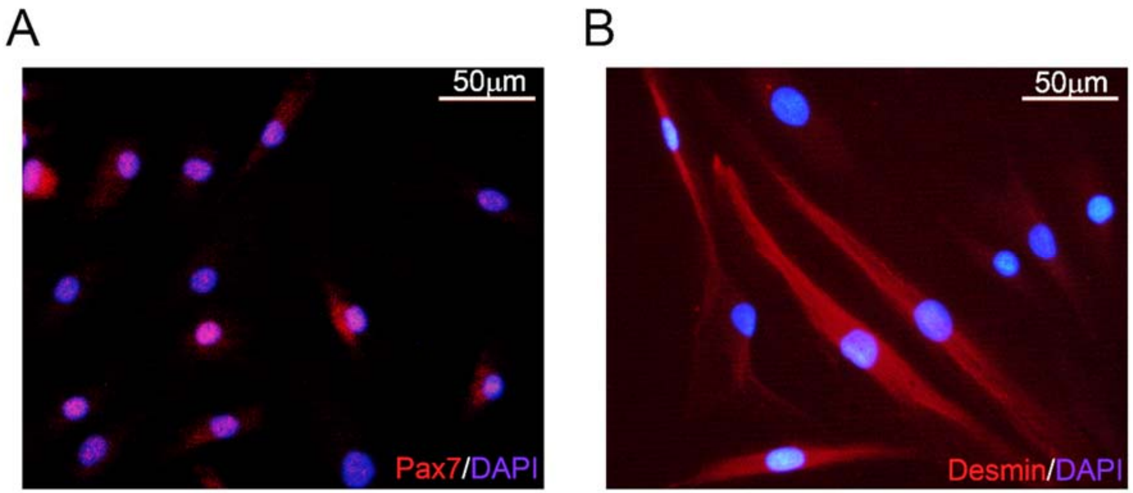

Sup Fig 1. Identification of human skeletal muscle mesenchymal progenitor cells (hSM-MPC). A. Pax7/DAPI co-stained hSM-MPC cells before differentiation; B. Desmin/DAPI co-stained hSM-MPC cells after differentiation. (400×).

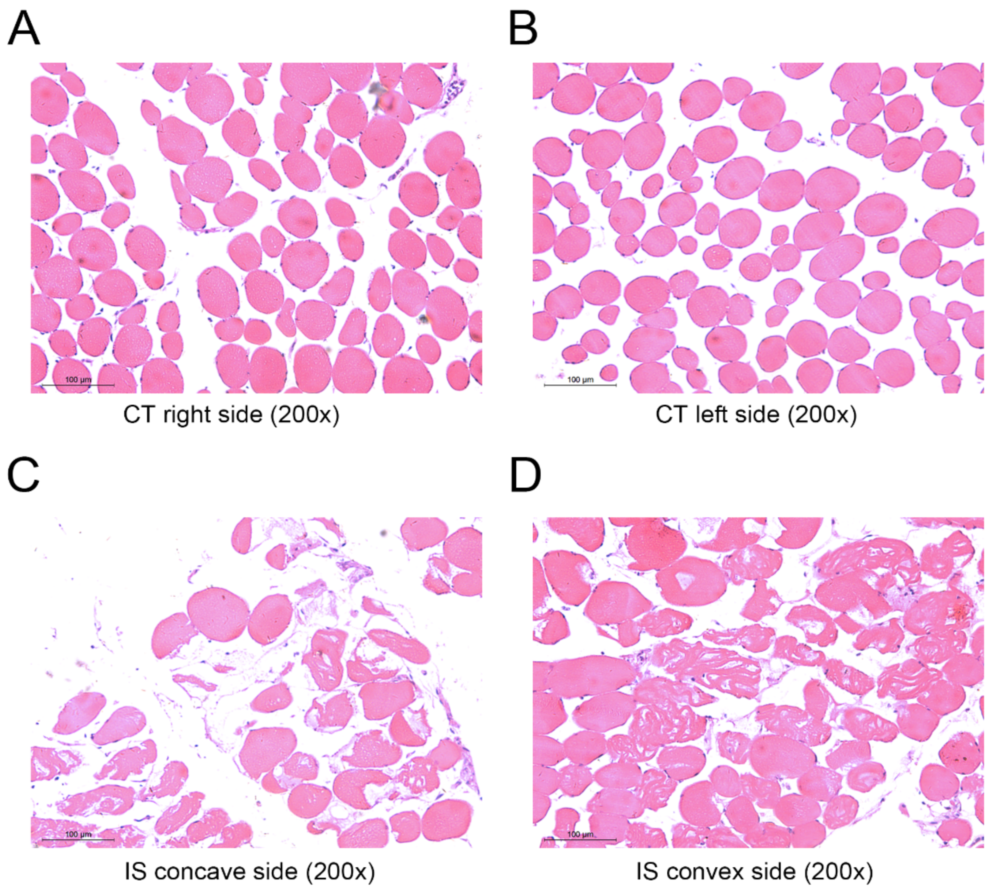

Sup Fig 2. H&E stained two sides of paraspinal muscles in IS patients and control group.

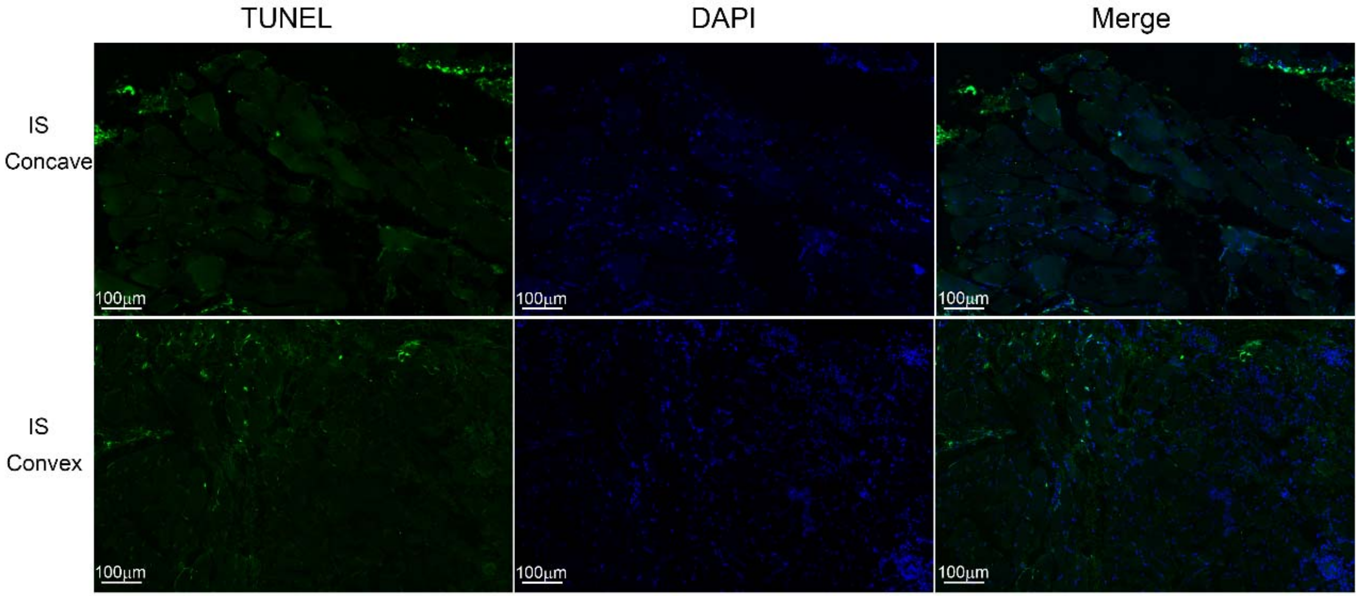

Sup Fig 3. TUNEL stained concave and convex sides of paraspinal muscle sections in IS patients (200×).

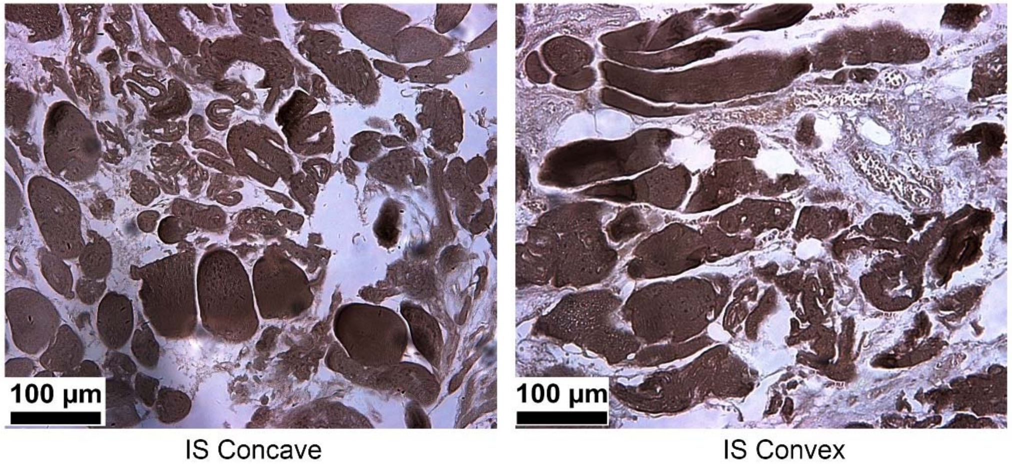

Sup Fig 4. ATPase stained concave and convex sides of paraspinal muscle sections in IS patients (200×).
